# Supplementary material for: Entorhinal grid-like codes for visual space during memory formation
Source: Nat Commun. 2025 Oct 20;16:9247. doi: 10.1038/s41467-025-64307-z (PMC12537863; doi:10.1038/s41467-025-64307-z)
Supplement: Supplementary file 1 — Supplementary Information [file 41467_2025_64307_MOESM1_ESM.pdf]

Supplementary Information:

Entorhinal grid-like codes for visual space during memory formation

Luise P. Graichen, Magdalena S. Linder, Lars Keuter, Ole Jensen, Christian F. Doeller, Claus Lamm, Tobias Staudigl, Isabella C. Wagner

Supplementary Results

S1: Association between total saccade numbers (saccade frequency) and recognition memory performance (*d*-prime) in the Vienna study

In the Donders study, we found that individual recognition memory performance (*d*-prime) was coupled to the total number of saccades that participants made when viewing later-remembered scene images. To test whether this was also the case in the Vienna study, we repeated the correlation analysis (number of saccades × *d*-prime from immediate and delayed tests, respectively). Indeed, we found a significant association between total saccade numbers and *d*-prime values from the immediate test ($N = 46$, $r_{Pearson} = 0.46$, 95% confidence interval (CI) = [0.20, 0.66], $p_{two\text{-}tailed} = 0.001$; **Supplementary Fig. S1**). Total saccade numbers and *d*-prime values from the delayed test period (carried out in addition to the immediate test one week later, s. Methods) were also positively correlated ($N = 46$, $r_{Pearson} = 0.50$, 95% CI = [0.24, 0.69], $p_{two\text{-}tailed} < 0.001$).

S2: Donders study, control analysis: No significant saccade-based grid-like codes in control regions

Across two independent studies, we found significantly increased saccade-based grid-like codes in the left entorhinal cortex as participants viewed scene images they were later asked to recognize. Even though the entorhinal cortex is known to house grid cells and most findings of grid cells or associated grid-like codes have been reported in the entorhinal cortex, there is evidence for grid-like coding in other brain regions as well. To test if our findings were specific to the entorhinal cortex, we analyzed grid-like codes in a set of control regions that are known to be involved in memory and visuo-oculomotion, including the hippocampus, the anterior thalamus, the FEF, and the visual cortex (**Methods**). We observed no significant saccade-based grid-like codes in any of these regions ($N = 29$; hippocampus: mean ± SEM, 0.019 ± 0.028; FEF: mean ± SEM, 0.021 ± 0.038; visual cortex: mean ± SEM, 0.002 ± 0.072; Wilcoxon test, all $p_{one\text{-}tailed} > 0.05$), except for the anterior thalamus (but this result appeared driven by an outlier; $N = 29$; anterior thalamus: mean ± SEM, 0.168 ± 0.087; **Supplementary Fig. S2**).

S3A: Donders study, control analysis: Significant saccade-based grid-like codes when reversing estimation and test data sets

To show that our result was not driven by a specific data partitioning scheme, we repeated the main analysis but reversed the estimation and test data halves (i.e., we estimated grid-like codes in the second half of the data and tested grid-like codes in the first half). Results were virtually identical ($N = 29$; bilateral entorhinal cortex: mean ± SEM, 0.069 ± 0.029, Wilcoxon test, $V = 340$, $p_{one\text{-}tailed} = 0.004$, Cohen's $d = 0.45$; **Supplementary Fig. S3A**).

S3B: Donders study, control analysis: Significant saccade-based grid-like codes after controlling for differences in durations between saccade directions

The accurate estimation of grid orientations relies on the even distribution of saccade durations along the different directions on the computer screen. As such, it is important to determine any potential biases in the distribution of saccade durations as these could greatly impact the results (that is, if a participant never made saccades in a specific direction, it is not possible to accurately estimate the grid orientation for that direction). Thus, we partitioned saccades into different directional bins with 10°-spacing and calculated the average saccade duration (in ms) for each directional bin per participant. We observed a significant bias in saccade durations in 8 out of 29 participants (i.e., a main effect of directional bin, $p < 0.05$ for 8/29). To mitigate this, we used an iterative process, and for each iteration randomly excluded 10% of the saccades until we could ensure that there was no significant difference across saccade durations between the different directional bins. Following this, we repeated the main analysis in these 8 participants (i.e., for each participant, we determined saccade-based grid-like codes using this reduced set of saccades) and performed the group analysis once more. The main result of bilateral entorhinal grid-like codes remained significant ($N = 29$, mean ± SEM, 0.109 ± 0.041, Wilcoxon test, $V = 328.5$, $p_{one\text{-}tailed} = 0.008$, $d = 0.49$, **Supplementary Fig. S3B**).

S4: Donders study, control analysis: No significant association between saccade-based grid-like codes and entorhinal BOLD activation

To check whether significant saccade-based grid-like codes were linked to an overall increase in the entorhinal BOLD signal (thus, reflecting individual differences in the signal-to-noise ratio of the fMRI data), we correlated the magnitude of grid-like codes in the bilateral entorhinal cortex with the average BOLD signal. The result indicated a negative correlation but was not significant ($N = 29$, $r_{Pearson} = -0.33$, 95% CI = [-0.62, 0.04], $p_{two\text{-}tailed} = 0.083$, **Supplementary Fig. S4**.

S5: Donders study, control analysis: No significant association between *d*-prime and saccade durations, or entorhinal BOLD activation

First, to test whether the relationship between saccade-based grid-like codes and recognition memory performance (measured as *d*-prime) was linked to specific saccade patterns (e.g., that participants with higher *d*-prime produced shorter saccades, possibly leading to lower grid-like codes), we correlated individual *d*-prime values with average individual saccade durations (in ms). This correlation was not significant ($N = 29$, $r_{Pearson} = -0.07$, 95% CI = [-0.43, 0.30], $p_{two\text{-}tailed} = 0.717$; **Supplementary Fig. S5A**). Second, to show that the brain-behavior relationship was not driven by differences in entorhinal BOLD signal (e.g., that for participants with higher *d*-prime values, the BOLD signal was lower), we correlated individual *d*-prime values with the average individual BOLD signal. The resulting correlation was also not significant ($N = 29$, $r_{Pearson} = 0.32$, 95% CI = [-0.06, 0.61], $p_{two\text{-}tailed} = 0.094$; **Supplementary Fig. S5B**).

S6: Donders study: No significant association between entorhinal grid-like codes and saccade characteristics

To assess whether the negative relationship between saccade-based entorhinal grid-like codes and recognition memory performance could be attributed to individual differences in saccade characteristics (e.g., participants with better recognition memory might have been able to memorize the scene images by producing saccades with shorter durations, potentially resulting in lower grid-like codes), we extracted the average saccade frequency (average saccade numbers per trial), velocity (average saccade speed across all trials in degrees of visual angle per second), and amplitude (average saccade distance across all trials in degrees of visual angle) per individual. We then correlated these measures with the saccade-based grid-like codes obtained from our main analysis (Donders study, bilateral entorhinal cortex, $N = 29$).

Results did not confirm a significant relationship between grid-like codes and average saccade frequency, velocity, or amplitude (**Supplementary Fig. S6**), indicating that individual differences in saccade characteristics did not account for the observed grid-like codes.

S7: Donders study: No significant association between entorhinal grid-like codes and visual exploration

Next, we conducted a more detailed analysis of visual exploration. Eye movements play a crucial role in memory formation, as they allow us to actively sample information from the environment for later recall. Higher numbers of saccades and fixations have been linked to better recognition memory across participants (Fehlmann et al., 2020; Loftus, 1972). Similarly, returns to previously viewed locations (so-called "revisitations") have been linked to better recognition memory and may support the encoding process by redirecting attention to specific elements of a given scene (Kragel et al., 2021). We reasoned that grid-like codes might be particularly linked to revisitations, supporting the encoding of translational vectors between salient stimulus features, akin to relational processing (Bicanski & Burgess, 2019; Killian & Buffalo, 2018).

To test whether these visual exploration patterns might have contributed to our results, we quantified the fixation and revisitation numbers per trial (see Supplementary Methods S2 for details). We first examined whether average fixation and revisitation frequencies were related to recognition memory performance (*d*-prime) across participants (Donders data set, $N = 32$), but this was not the case (**Supplementary Fig. S7A**). Average fixation and revisitation frequencies were not significantly associated with the magnitude of saccade-based entorhinal grid-like codes (Donders data set, $N = 29$, all $p > 0.05$, **Supplementary Fig. S7B**). Overall, these findings show that potential differences in visual exploration between individuals did not significantly impact our results.

S8: Saccade-based grid-like codes and memory durability in the Vienna study

In the Vienna study, recognition memory performance was measured twice, immediately after the study period (immediate test) and one week later (delayed test). Thus, the Vienna data allowed us to examine recognition memory performance at both test time points (immediate and delayed *d*-prime), as well as overall memory durability. Participants' *d*-prime values in the immediate and delayed tests were positively correlated ($N = 20$, $r_{Pearson} = 0.87$, 95% CI = [0.690, 0.947], $p_{two\text{-}tailed} < 0.0001$). To test whether grid-like codes were tied to memory durability, we quantified a memory durability score (Wagner et al., 2019) that yielded the proportion of durable memories (remembered in both immediate and delayed tests) with respect to weak memories (remembered in the immediate test but forgotten in the delayed test; durable/weak). Consistent with the above, there was no significant relationship between saccade-based grid-like coding in the left entorhinal cortex and individual memory durability scores ($N = 20$, $r_{Pearson} = 0.22$, 95% CI = [-0.246, 0.604], $p_{two\text{-}tailed} = 0.350$).

Supplementary Methods

S1: Assessment of eye tracking data quality in the Donders and the Vienna study

To validate the quality of our eye tracking data, we assessed the distribution of saccade directions, the relationship between saccade amplitudes and velocities, fixation frequencies and durations, as well as calibration accuracy across subjects.

Distribution of saccade directions:

To examine potential biases in saccade directions, we visualized the angular distribution (in degrees) of all saccades that occurred during the study period (**Supplementary Fig. S8**). The distribution depicts the number of saccades in each direction (0° = rightward, 90° = upward, 180° = leftward, 270° = downward). In the Donders study, saccades were primarily oriented along the horizontal axis. In the Vienna study, a similar pattern is seen, albeit less pronounced, with more evenly distributed saccades across all cardinal directions. This slight deviation likely reflects differences in the stimulus presentation format. The rectangular scene images presented in the Donders study (640 × 480) may have encouraged more horizontal saccades compared to the square scene images in the Vienna study (500 × 500). Overall, participants' gaze behavior aligns with typical patterns observed during free viewing of visual scene stimuli (Foulsham et al., 2008).

Saccade amplitudes against velocities:

Next, we plotted saccade amplitudes against saccade velocities (**Supplementary Fig. S8**). In healthy individuals, these measures are typically positively correlated, reflecting the principle that larger saccades tend to be faster (Bahill et al., 1975; Gibaldi & Sabatini, 2021). Consistent with this, we observed a positive correlation across both studies, indicating reliable saccade measurements despite the challenges imposed by the fMRI environment (Donders study: $N = 32$, $r_{Pearson} = 0.891$, 95% CI = [0.787, 0.946], $p_{two\text{-}tailed} < .001$; Vienna study: $N = 46$, $r_{Pearson} = .532$, 95% CI = [.286, .712], $p_{two\text{-}tailed} < .001$).

Average fixation frequency and duration:

The main focus of our study was to investigate whether participants exhibited grid-like coding based on saccades that occurred during the study period and if this would subserve memory formation. Accordingly, our primary assessment of eye data quality focused on saccade measures. To also validate the quality of eye fixations, we returned to the identification of fixations as performed by the EyeLink system, where fixations were defined as periods between saccades and blinks. Fixations shorter than 100 ms were excluded to ensure that only meaningful fixation events were considered (Hannula, 2010). The average number of fixations per trial across subjects (Donders study: $N = 32$, mean ± SEM, 4.50 ± .51; Vienna study: $N = 46$, 6.00 ± .26) and the average fixation durations per subject (Donders study: $N = 32$, mean ± SEM, 184.99 ± 4.47 ms; Vienna study: $N = 46$, 210.78 ± 1.75 ms) were within the expected range for free viewing of visual scene stimuli (e.g., Nuthmann, 2017), further supporting the validity of our eye tracking data (**Supplementary Fig. S8**).

Calibration accuracy:

Finally, we examined calibration accuracy as an additional indicator of data quality. Each recording session began with a calibration-validation procedure according to the standard EyeLink protocol. To map raw eye movement data onto screen coordinates, participants first sequentially fixated on nine targets arranged in a 3 × 3 grid (calibration). In a second step, participants were asked to fixate the same targets once more until the differences between the current and the previously obtained gaze positions were < 1° of visual angle (validation). If these criteria were met, the eye tracker recording was started.

The average calibration error across all participants in the Vienna study (*N* = 46, mean ± SEM, 0.596 ± 0.028 degrees of visual angle, dva, **Supplementary Fig. S9**) confirmed that calibration accuracy remained below the 1° threshold. The two participants who exceeded this threshold were excluded from the grid-like code analysis and, therefore, did not influence the reported results (**Supplementary Fig. S9, right panel**). As our analyses primarily relied on gaze direction rather than absolute gaze position, calibration accuracy was less critical for our main findings.

S2: Quantification of revisitations in the Donders study

To quantify revisitations (i.e., returns to previously fixated locations), we first defined fixations as periods between saccades and blinks, as identified by the EyeLink system. Saccade detection was based on velocity and acceleration thresholds, which were automatically adjusted for each participant based on the signal-to-noise ratio during calibration. The average threshold values were 78.2°/s for velocity and 241.9°/s^2 for acceleration. To ensure consistent and meaningful fixation detection across participants, we excluded fixations with a duration shorter than 100 ms (Hannula, 2010). In a next step, we quantified the number of revisitations per trial. We applied the “CalcRets” function from the “PyEyeSim” package (https://github.com/jozsarato/PyEyeSim), which identified fixations that returned to a previously visited location within a threshold of 1° of visual angle. To ensure that revisitations reflected actual return movements rather than incidental proximity to a previously fixated location, the current fixation was required to be closer to the revisited location than the immediately preceding fixation.

Supplementary Discussion

Entorhinal grid-like codes in relation to saccade characteristics and visual exploration patterns

A possible explanation for the negative brain-behavior result is that memory success was associated with differences in eye movements during encoding. For instance, participants with better recognition memory might have been able to memorize the scene images by making saccades with shorter durations, resulting in lower grid-like codes. While this was not the case, we found subtle trends of higher grid-like codes being associated with lower average saccade frequencies, velocities, and amplitudes (**Supplementary Fig. S6**). We encourage future studies to test the extent to which grid-like codes are tied to saccade characteristics. Memory performance might also be influenced by the number of fixations or revisitations. The latter were previously linked to better recognition memory performance and may support thorough encoding by redirecting attention to specific elements of a scene (Kragel et al., 2021). We could not detect a significant relationship between grid-like codes, fixations, revisitations, or recognition memory performance (**Supplementary Fig. S7A**, **Supplementary Fig. S7B**). To further elucidate the relationship between grid-like codes and memory, future studies may examine the association with the reinstatement of eye movements during retrieval (Johansson et al., 2022). Overall, these findings reflect some inter-individual variability in visual exploration but did not appear to significantly contribute to the observed grid-like codes.

Supplementary Figures

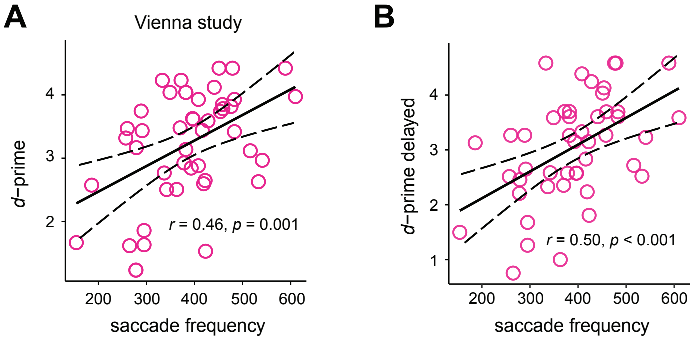

Fig. S1: Vienna study: Recognition memory performance and saccades.

A: The scatter plot displays the positive correlation between total saccade numbers (saccade frequency) and recognition memory performance (*d*-prime) per participant for the Vienna study (Pearson correlation, two-tailed, $N = 46$, $p = 0.001$). B: Total saccade numbers (saccade frequency) and *d*-prime from the delayed test were also positively correlated (Pearson correlation, two-tailed, $N = 46$, $p = 0.0004$). Regression fit is indicated by the solid lines, and confidence intervals (CI 95%) are marked by the dashed lines. Source data are provided as a Source Data file.

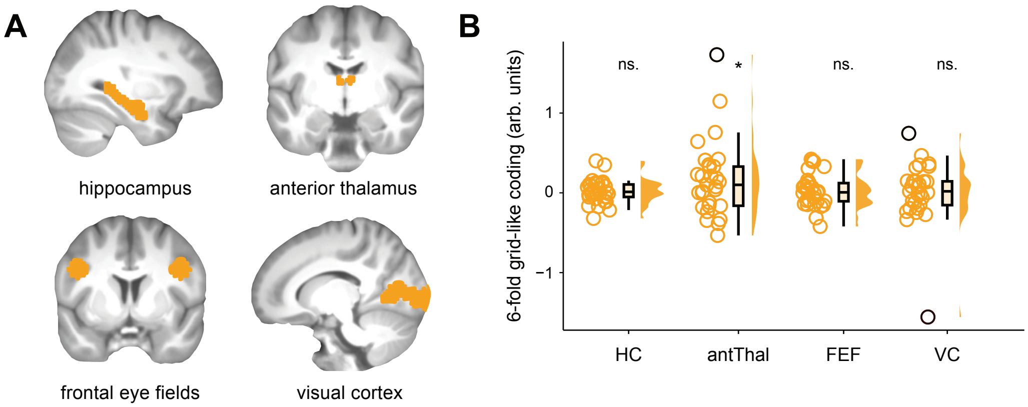

Fig. S2: Donders study: No significant saccade-based grid-like codes in control regions.

A: To determine whether saccade-based grid-like codes were significantly increased in other regions apart from the entorhinal cortex, we defined four additional regions of interest (ROIs; projected in orange onto the normalized T1-weighted structural scan), the hippocampus (HC), the anterior thalamus (antThal), the FEF, and the visual cortex (VC). B: We did not detect significant grid-like codes in any of these control regions (arb. units = arbitrary units; Wilcoxon tests, one-tailed, $N = 29$, $p > 0.05$), except for the anterior thalamus (but this result appeared driven by an outlier; before outlier removal: one-tailed, $N = 29$, $V = 295$, p-value = 0.048, $d = 0.356$; after outlier removal: one-tailed, $N = 28$, $V = 266$, p-value = 0.078). Data points show individual grid-like codes during study periods for each control region, and boxplots show the median (upper and lower borders mark the interquartile range, whiskers show minimum and maximum non-outlier values. Unadjusted p-values are reported, with effects below $p < 0.01$ also meeting the Bonferroni-corrected significance criterion (s. Methods). Outliers (± 3 × MAD) are marked in black, *= significant at $p < 0.5$, ns. = not significant. Source data are provided as a Source Data file.

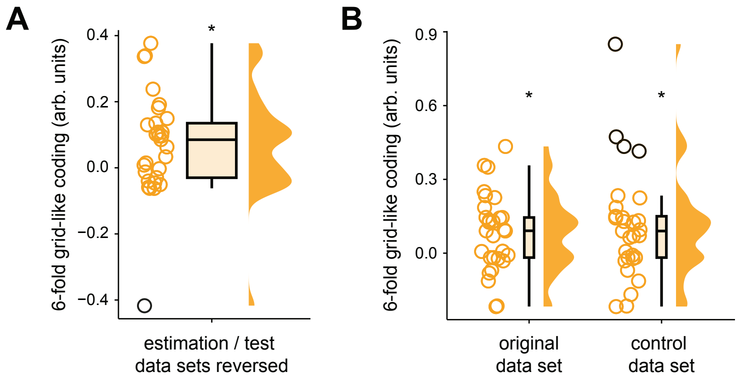

Fig. S3: Donders study: Significant saccade-based grid-like codes when reversing estimation and test data sets, and after controlling for differences in saccade durations.

A: When repeating the main analysis for reversed estimation and test data halves, we again found significant saccade-based grid-like coding (arb. units = arbitrary units; Wilcoxon tests, one-tailed, N = 29, p = 0.004, d = 0.45). B: After iteratively excluding 10% of saccades to control for individual differences in saccade durations, we observed significant grid-like coding in the bilateral entorhinal cortex (original data set: one-tailed, N = 29, p = 0.006, d = 0.53; reduced data set: one-tailed, N = 29, p = 0.008, d = 0.49). Data points show individual grid-like codes during study periods, and boxplots show the median (upper and lower borders mark the interquartile range, whiskers show minimum and maximum non-outlier values. Outliers (± 3 × MAD) are shown in black. * = significant at $p < 0.05$. Source data are provided as a Source Data file.

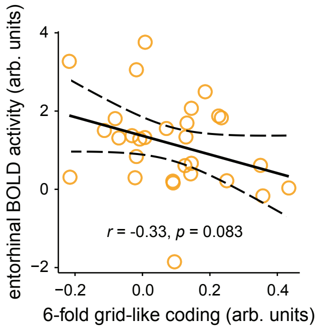

Fig. S4: Donders study: No significant association between saccade-based grid-like codes and entorhinal BOLD activation.

Control analysis showing that our main result of significant saccade-based grid-like coding was not driven by changes in entorhinal BOLD signal (arb. units = arbitrary units; Pearson correlation, two-tailed, N = 29, p = 0.083). The regression fit is indicated by the solid line, and the confidence interval (95% CI) is marked by the dashed lines. Source data are provided as a Source Data file.

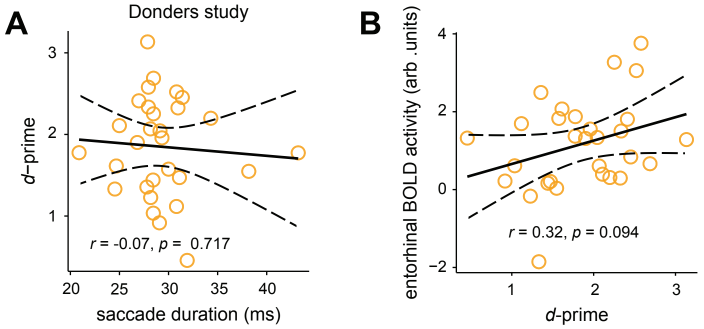

Fig. S5: Donders study: No significant association between *d*-prime and saccade durations, or entorhinal BOLD activation.

A: There was no significant correlation between the mean saccade durations and *d*-prime values across participants in the Donders study (Pearson correlation, two-tailed, N = 29, p = 0.717). B: Higher *d*-prime values were not driven by variations in the overall BOLD signal across participants (arb. units = arbitrary units; Pearson correlation, two-tailed, N = 29, p = 0.094). Regression fit is indicated by the solid lines, and confidence intervals (95% CI) are marked by the dashed lines. Source data are provided as a Source Data file.

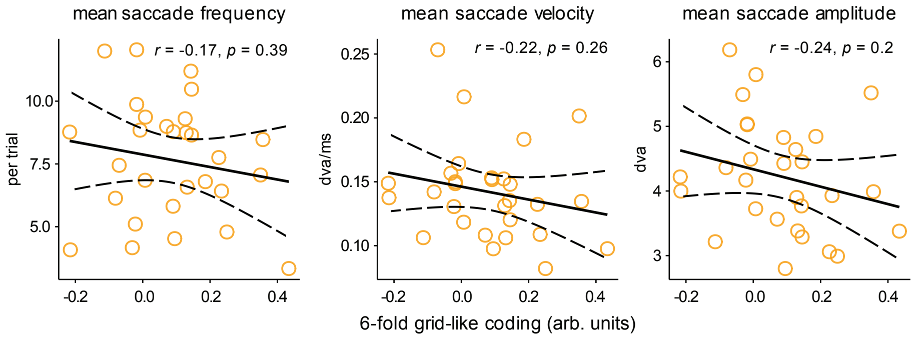

Fig. S6: Subtle trend toward higher saccade-based grid-like codes at less frequent, slower, and shorter saccades.

The scatter plots show Pearson correlations (two-tailed, N = 29) between the magnitude of saccade-based entorhinal grid-like codes (arb. units = arbitrary units) and saccade characteristics such as the mean frequency per trial, and the mean velocity and amplitude across trials (dva = degree of visual angle). Results are not significant (all $p > .05$) but suggest a subtle trend toward a negative relationship. Regression fit is indicated by the solid lines, and confidence intervals (95% CI) are marked by the dashed lines. Source data are provided as a Source Data file.

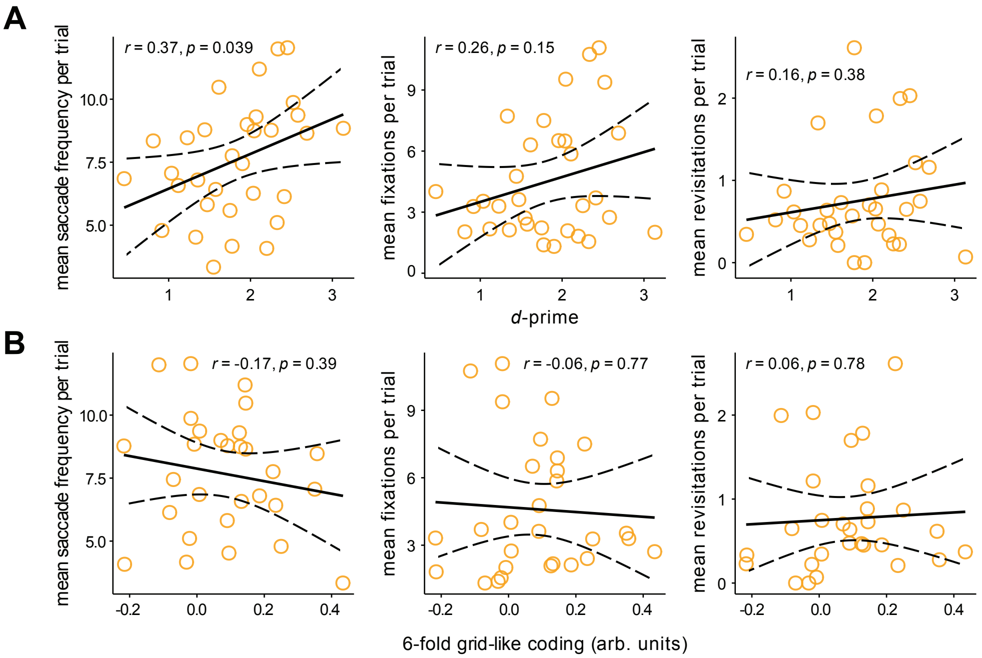

Fig. S7: Individual differences in visual exploration in relation to recognition memory performance (*d*-prime) and saccade-based entorhinal grid-like coding.

The scatter plots display (A) Pearson correlations between visual exploration patterns (saccades, fixations, revisitations) and recognition memory performance (*d*-prime; two-tailed, $N = 32$). Higher mean saccade frequency per trial was associated with higher *d*-prime. Other results were not significant (all $p > 0.05$) but indicated a consistent trend toward a positive relationship. (B) Pearson correlations between visual exploration patterns and saccade-based entorhinal grid-like coding (arb. units = arbitrary units; two-tailed, $N = 29$). Results were not significant (all $p > 0.05$). Regression fit is indicated by the solid lines, and confidence intervals (95% CI) are marked by the dashed lines. Source data are provided as a Source Data file.

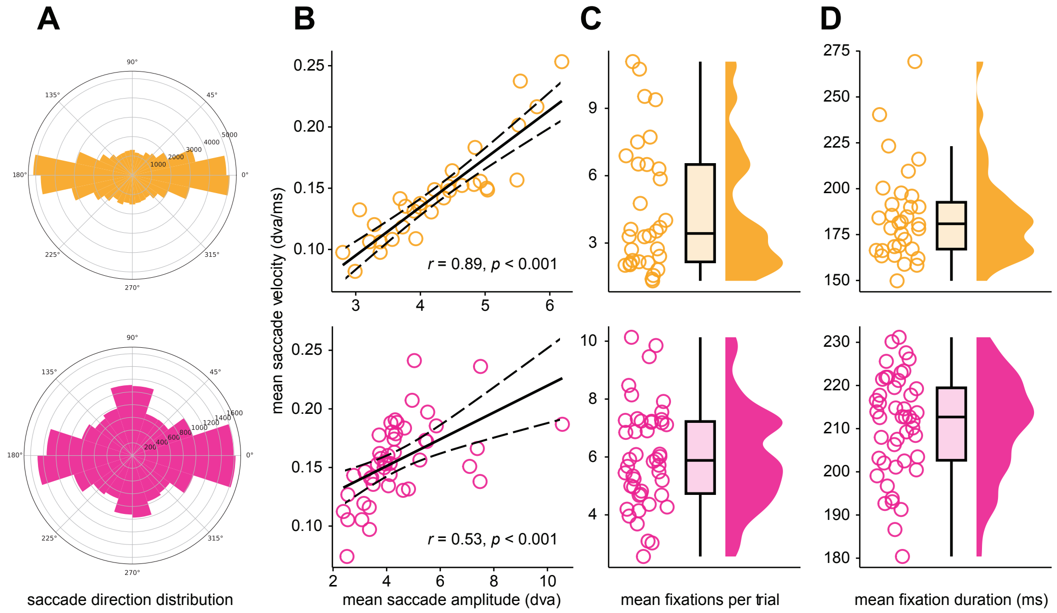

Fig. S8: Quality assessment of eye movement data during scene encoding.

A: Polar histogram showing the distribution of saccade directions across all trials, reflecting typical patterns of visual exploration during scene encoding (Donders: $N = 32$; Vienna: $N = 46$). B: Scatter plots show Pearson correlations between saccade amplitude (dva = degree of visual angle) and velocity (dva/ms) across participants (two-sided, Donders: $N = 32$, $p < 0.001$; Vienna: $N = 46$, $p < 0.001$). Regression fit is indicated by the solid lines, and confidence intervals (95% CI) are marked by the dashed lines. C: Mean fixations per trial across participants (Donders: $N = 32$; Vienna: $N = 46$). D: Mean fixation durations (ms) across participants (Donders: $N = 32$; Vienna: $N = 46$). Data points show values per subject, boxplots show the median (upper and lower borders mark the interquartile range, whiskers show minimum and maximum non-outlier values), orange = Donders study, pink = Vienna study. Source data are provided as a Source Data file.

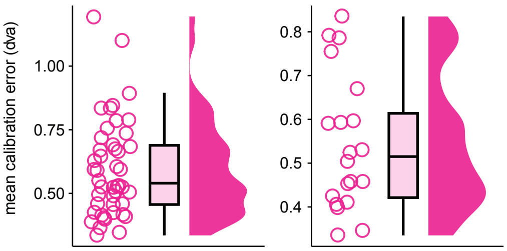

Fig. S9: Calibration accuracy.

Left panel: Distribution of mean calibration error (dva = degree of visual angle) across all participants in the Vienna study ($N = 46$: mean ± SEM, 0.596 ± 0.028 dva), right panel: Distribution of mean calibration error (dva) for the subset of participants included in the grid-like coding analysis ($N = 20$: mean ± SEM, 0.543 ± 0.034 dva). Values remained below the 1° threshold, confirming sufficient calibration accuracy. Data points show values per subject, boxplots show the median (upper and lower borders mark the interquartile range, whiskers show minimum and maximum non-outlier values). Source data are provided as a Source Data file.

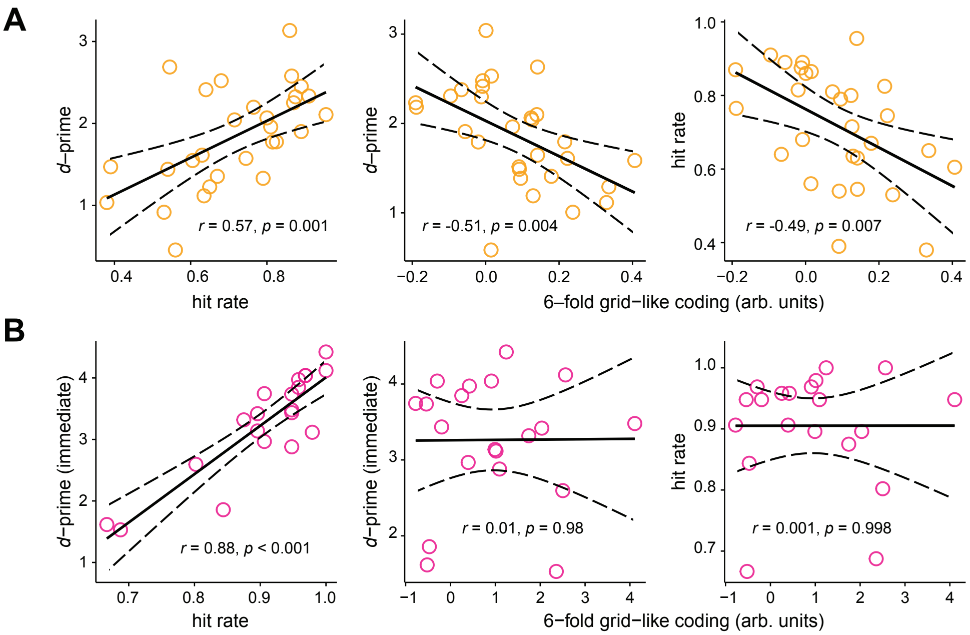

Fig. S10.: Comparison between *d*-prime and hit rate.

A: Donders study, the scatter plots show Pearson correlations (two-tailed, $N = 29$) between *d*-prime and hit rate (left panel), grid-like coding (arb. units = arbitrary units, bilateral entorhinal cortex) and *d*-prime (middle panel), and grid-like coding and hit rate (right panel). B: Vienna study, the scatter plots show Pearson correlations (two-tailed, $N = 20$) between *d*-prime and hit rate (left panel), grid-like coding (left entorhinal cortex) and *d*-prime (middle panel), and grid-like coding and hit rate (right panel). Regression fit is indicated by the solid lines, and confidence intervals (95% CI) are marked by the dashed lines. The results illustrate the close relationship between the two behavioral metrics, yielding virtually identical results. Source data are provided as a Source Data file.

Supplementary Tables

Table 1

Grid-like coding during hits and misses (Wilcoxon tests)

Study	Condition	*N*	Mean ± SEM	Wilcoxon *V*	$p_{\text{one-tailed}}$	Cohen's *d*	Wilcoxon's *r*
Donders	Hits	26	.119 ± .054	237.5	.023	.43	.40
Donders	Misses	26	.050 ± .126	201	.26	.08	.13
Vienna	Hits	20	1.074 ± .315	181	.002	.76	.63
Vienna	Misses	9	-3.302 ± 3.377	17	.75	-.23	.22

Note. Grid-like coding is reported as mean ± SEM. Cohen's *d* and Wilcoxon's *r* reflect effect sizes. All *p*-values are one-tailed.

Table 2

Correlations between grid-like coding and d-prime

Study	Condition	*N*	*r*	95% CI	$p_{\text{two-tailed}}$
Donders	Hits	26	-.12	[-.483, .282]	.57
Donders	Misses	26	-.24	[-.573, .163]	.24
Vienna	Hits	20	-.36	[-.690, 0.102]	.12
Vienna	Misses	9	.41	[-.352, .843]	.28

Note. Pearson's *r* values reflect the correlation between grid-like coding and recognition memory performance (*d*-prime). All *p*-values are two-tailed.

Table 3

Correlations between grid-like coding and hit rate

Study	Condition	*N*	*r*	95% CI	$p_{\text{two-tailed}}$
Donders	Hits	26	-.44	[-.704, -.059]	.03
Donders	Misses	26	-.07	[-.444, .328]	.74
Vienna	Hits	20	-.44	[-.736, .009]	.05
Vienna	Misses	9	.12	[-.591, .726]	.76

Note. Pearson's *r* values reflect the correlation between grid-like coding and recognition memory performance (hit rate). All *p*-values are two-tailed.

Supplementary References

Bahill, A. T., Clark, M. R., & Stark, L. (1975). The main sequence, a tool for studying human eye movements. *Mathematical Biosciences*, *24*(3), 191–204. https://doi.org/10.1016/0025-5564(75)90075-9

Bicanski, A., & Burgess, N. (2019). A Computational Model of Visual Recognition Memory via Grid Cells. *Current Biology*, *29*(6), 979-990.e4. https://doi.org/10.1016/J.CUB.2019.01.077

Fehlmann, B., Coynel, D., Schicktanz, N., Milnik, A., Gschwind, L., Hofmann, P., Papassotiropoulos, A., & De Quervain, D. J.-F. (2020). Visual Exploration at Higher Fixation Frequency Increases Subsequent Memory Recall. *Cerebral Cortex Communications*, *1*(1), tgaa032. https://doi.org/10.1093/texcom/tgaa032

Foulsham, T., Kingstone, A., & Underwood, G. (2008). Turning the world around: Patterns in saccade direction vary with picture orientation. *Vision Research*, *48*(17), 1777–1790. https://doi.org/10.1016/j.visres.2008.05.018

Gibaldi, A., & Sabatini, S. P. (2021). The saccade main sequence revised: A fast and repeatable tool for oculomotor analysis. *Behavior Research Methods*, *53*(1), 167–187. https://doi.org/10.3758/s13428-020-01388-2

Hannula, D. E. (2010). Worth a glance: Using eye movements to investigate the cognitive neuroscience of memory. *Frontiers in Human Neuroscience*, *4*. https://doi.org/10.3389/fnhum.2010.00166

Johansson, R., Nyström, M., Dewhurst, R., & Johansson, M. (2022). Eye-movement replay supports episodic remembering. *Proceedings of the Royal Society B*, *289*(1977), 20220964.

Killian, N. J., & Buffalo, E. A. (2018). Grid cells map the visual world. *Nature Neuroscience*, *21*(2), 161–162. https://doi.org/10.1038/s41593-017-0062-4

Kragel, J. E., Schuele, S., VanHaerents, S., Rosenow, J. M., & Voss, J. L. (2021). Rapid coordination of effective learning by the human hippocampus. *SCIENCE ADVANCES*.

Loftus, G. R. (1972). Eye fixations and recognition memory for pictures. *Cognitive psychology*, *3*(4), 525–551.

Nuthmann, A. (2017). Fixation durations in scene viewing: Modeling the effects of local image features, oculomotor parameters, and task. *Psychonomic Bulletin & Review*, *24*(2), 370–392. https://doi.org/10.3758/s13423-016-1124-4

Wagner, I. C., Van Buuren, M., & Fernández, G. (2019). Thalamo-cortical coupling during encoding and consolidation is linked to durable memory formation. *NeuroImage*, *197*, 80–92. https://doi.org/10.1016/j.neuroimage.2019.04.055
