# Supplementary material for: Entorhinal grid-like codes for visual space during memory formation
Source: Nat Commun. 2025 Oct 20;16:9247. doi: 10.1038/s41467-025-64307-z (PMC12537863; doi:10.1038/s41467-025-64307-z)
Supplement: Supplementary file 3 — Reporting Summary [file 41467_2025_64307_MOESM3_ESM.pdf]

nature portfolio

Corresponding author(s): Isabella C. Wagner; Luise P. Graichen

Last updated by author(s): 2025/08/26

Reporting Summary

Nature Portfolio wishes to improve the reproducibility of the work that we publish. This form provides structure for consistency and transparency in reporting. For further information on Nature Portfolio policies, see our Editorial Policies and the Editorial Policy Checklist.

Statistics

For all statistical analyses, confirm that the following items are present in the figure legend, table legend, main text, or Methods section.

n/a	Confirmed	
☐	☒	The exact sample size (*n*) for each experimental group/condition, given as a discrete number and unit of measurement
☐	☒	A statement on whether measurements were taken from distinct samples or whether the same sample was measured repeatedly
☐	☒	The statistical test(s) used AND whether they are one- or two-sided<br>*Only common tests should be described solely by name; describe more complex techniques in the Methods section.*
☐	☒	A description of all covariates tested
☐	☒	A description of any assumptions or corrections, such as tests of normality and adjustment for multiple comparisons
☐	☒	A full description of the statistical parameters including central tendency (e.g. means) or other basic estimates (e.g. regression coefficient) AND variation (e.g. standard deviation) or associated estimates of uncertainty (e.g. confidence intervals)
☐	☒	For null hypothesis testing, the test statistic (e.g. *F*, *t*, *r*) with confidence intervals, effect sizes, degrees of freedom and *P* value noted<br>*Give P values as exact values whenever suitable.*
☒	☐	For Bayesian analysis, information on the choice of priors and Markov chain Monte Carlo settings
☒	☐	For hierarchical and complex designs, identification of the appropriate level for tests and full reporting of outcomes
☐	☒	Estimates of effect sizes (e.g. Cohen's *d*, Pearson's *r*), indicating how they were calculated

Our web collection on statistics for biologists contains articles on many of the points above.

Software and code

Policy information about availability of computer code

Data collection	Study 1 (Donders study): Imaging data were collected at the Donders Institute for Brain, Cognition and Behaviour (Nijmegen, The Netherlands), using a 3T Prisma Fit scanner (Siemens, Erlangen, Germany) equipped with a 32-channel head coil.<br><br>Study 2 (Vienna study): Imaging data were collected at the Neuroimaging Center of the University of Vienna, using a 3T Skyra MR-Scanner (Siemens, Erlangen, Germany) equipped with a 32-channel head coil.
Data analysis	Data was analyzed with openly available software code including SPM (software versions 8 and 12, https://www.fil.ion.ucl.ac.uk/spm/), Functional Magnetic Resonance Imaging of the Brain (FMRIB) Software Library (FSL; software version 5.0.1; https://fsl.fmrib.ox.ac.uk/fsl), Grid Code Analysis Toolbox (GridCAT, software version 1.0.4, https://www.nitrc.org/projects/gridcat), Fieldtrip (latest software version downloaded on 4th March, updated daily; https://www.fieldtriptoolbox.org), Automatic Segmentation of Hippocampal Subfields algorithm (software version 1.0.0, https://sites.google.com/site/hipposubfields/), MATLAB (The Mathworks, Natick, MA, USA, R2020b), R (software version 4.3.0; https://www.r-project.org). Custom code is openly available at the Open Science Framework: https://osf.io/vp7t3/.

For manuscripts utilizing custom algorithms or software that are central to the research but not yet described in published literature, software must be made available to editors and reviewers. We strongly encourage code deposition in a community repository (e.g. GitHub). See the Nature Portfolio guidelines for submitting code & software for further information.

Data

Policy information about availability of data

All manuscripts must include a data availability statement. This statement should provide the following information, where applicable:

- Accession codes, unique identifiers, or web links for publicly available datasets
- A description of any restrictions on data availability
- For clinical datasets or third party data, please ensure that the statement adheres to our policy

Donders study: Raw, anonymized data will be made publicly available upon completion of orthogonal projects that rely on the same data set. Until then, raw data are available upon request to the corresponding authors (isabella.wagner@univie.ac.at, luise.philine.graichen@univie.ac.at) in accordance with the requirements of the institute, the funding body, and the institutional ethics board.

Vienna study: Raw, anonymized fMRI and eye tracking data are available upon request to the corresponding authors (isabella.wagner@univie.ac.at, luise.philine.graichen@univie.ac.at). At present, participant informed consent does not allow for depositing the full data set.

Source data to reproduce figures and tables of both studies (behavioral performance, ROI-based grid-like code results, and (un-)thresholded statistical whole-brain fMRI maps) are openly available at the Open Science Framework (https://osf.io/vp7t3/). Source Data are provided with this paper.

Research involving human participants, their data, or biological material

Policy information about studies with human participants or human data. See also policy information about sex, gender (identity/presentation), and sexual orientation and race, ethnicity and racism.

Reporting on sex and gender	Study 1 (Donders study): 23 females and 9 males participated in the study.<br>Study 2 (Vienna study): 15 females and 5 males participated in the study.<br>Biological sex was determined by self-report. Sex differences were not the focus of this study, and sex-based analyses were not performed. We propose that the findings of this study apply to both sexes.
Reporting on race, ethnicity, or other socially relevant groupings	Measures of race, ethnicity, and other socially relevant groupings were not included in our data collection.
Population characteristics	Study 1 (Donders study):<br>Forty-eight participants volunteered for this study. Sixteen participants were excluded due to not completing the study (7 individuals), excessive motion (4 individuals), technical problems during the data recording (3 individuals), a low number of identified saccades during the fMRI session (1 individual), or low recognition memory performance during the MRI session (1 individual). The final sample thus comprised 32 participants (23 females, age range 18-30 years, mean age = 23 years, 32 right-handed). All individuals were healthy and did not report any history of neurological and/or psychiatric disorders, had normal or corrected-to-normal vision, and provided written informed consent before the start of the experiment.<br><br>Study 2 (Vienna study):<br>Fifty participants were invited to partake in the study. Four participants were excluded due to technical problems during the eye tracking recording, leaving a sample of 46 individuals. For the analysis of grid-like codes, 26 more participants were excluded due to a low number of identified saccades during the fMRI session or due to signal drop-outs in the entorhinal cortex region, resulting in a sample of 20 participants (15 females, age range 18-29 years, mean age = 21.75 years, 18 right-handed). All participants were healthy, did not report any history of neurological and/or psychiatric disorders, had normal, or corrected-to-normal vision, and provided written informed consent prior to participation.
Recruitment	Participants were selected solely based on their eligibility to participate in the study (health, normal or corrected-to-normal vision, eligibility for MRI). There was no self-selection bias in the recruitment.
Ethics oversight	Study 1 (Donders study):<br>The study was reviewed and approved by the local ethics committee (Commissie Mensgebonden Onderzoek, region Arnhem-Nijmegen, The Netherlands; reference number CMO-2014/288).<br><br>Study 2 (Vienna study):<br>The study was reviewed and approved by the ethics committee of the University of Vienna (reference number 00538).

Note that full information on the approval of the study protocol must also be provided in the manuscript.

Field-specific reporting

Please select the one below that is the best fit for your research. If you are not sure, read the appropriate sections before making your selection.

☒ Life sciences ☐ Behavioural & social sciences ☐ Ecological, evolutionary & environmental sciences

For a reference copy of the document with all sections, see nature.com/documents/nr-reporting-summary-flat.pdf

Life sciences study design

All studies must disclose on these points even when the disclosure is negative.

Sample size	Sample size calculations were based on fMRI studies that tested grid-like codes during (mental) navigation (pooled effect size, Cohen's d = 0.49; Doeller et al., 2010; Bellmund et al., 2016; Horner et al., 2016). Based on this medium sized effect, we would need a sample of 35 participants to detect grid-like codes with a power of 80% (α error probability = 0.05). To account for potential dropouts, we increased sample sizes to 48 (Donders study) and 50 participants (Vienna study).
Data exclusions	Study 1 (Donders study):<br>Sixteen participants were excluded due to not completing the study (7 individuals), excessive motion (4 individuals), technical problems during the data recording (3 individuals), a low number of identified saccades during the fMRI session (1 individual), or low recognition memory performance during the MRI session (1 individual). The final sample thus comprised 32 participants (23 females, age range 18-30 years, mean age = 23 years, 32 right-handed).<br><br>Study 2 (Vienna study):<br>Four participants were excluded due to technical problems during the eye tracking recording, leaving a sample of 46 individuals. For the analysis of grid-like codes, 26 more participants were excluded due to a low number of identified saccades during the fMRI session or due to signal drop-outs in the entorhinal cortex region, resulting in a sample of 20 participants (15 females, age range 18-29 years, mean age = 21.75 years, 18 right-handed).
Replication	Across two independent data sets (Donders and Vienna study), we found significantly increased saccade-based grid-like codes in the left entorhinal cortex while participants studied scene images, highlighting the replicability of our results.
Randomization	There were no experimental groups.
Blinding	Blinding of experimenters was not necessary, as there were no experimental groups.

Reporting for specific materials, systems and methods

We require information from authors about some types of materials, experimental systems and methods used in many studies. Here, indicate whether each material, system or method listed is relevant to your study. If you are not sure if a list item applies to your research, read the appropriate section before selecting a response.

Materials & experimental systems

n/a	Involved in the study
☒	☐ Antibodies
☒	☐ Eukaryotic cell lines
☒	☐ Palaeontology and archaeology
☒	☐ Animals and other organisms
☒	☐ Clinical data
☒	☐ Dual use research of concern
☒	☐ Plants

Methods

n/a	Involved in the study
☒	☐ ChIP-seq
☒	☐ Flow cytometry
☐	☒ MRI-based neuroimaging

Plants

Seed stocks	*Report on the source of all seed stocks or other plant material used. If applicable, state the seed stock centre and catalogue number. If plant specimens were collected from the field, describe the collection location, date and sampling procedures.*
Novel plant genotypes	*Describe the methods by which all novel plant genotypes were produced. This includes those generated by transgenic approaches, gene editing, chemical/radiation-based mutagenesis and hybridization. For transgenic lines, describe the transformation method, the number of independent lines analyzed and the generation upon which experiments were performed. For gene-edited lines, describe the editor used, the endogenous sequence targeted for editing, the targeting guide RNA sequence (if applicable) and how the editor was applied.*
Authentication	*Describe any authentication procedures for each seed stock used or novel genotype generated. Describe any experiments used to assess the effect of a mutation and, where applicable, how potential secondary effects (e.g. second site T-DNA insertions, mosiacism, off-target gene editing) were examined.*

Magnetic resonance imaging

Experimental design

Design type	Task design, event-related
Design specifications	Participants of both studies completed a recognition memory task (study period and test period) during fMRI scanning

while their eye movements were recorded.

Study 1 (Donders study):
During the study period, participants were instructed to memorize 200 scene images (100 indoor, 100 outdoor). Each scene was shown for 4 s during which participants could freely view the image. To ensure attention to each scene, participants were asked to judge whether the image depicted an indoor or outdoor scenario via button press during the subsequent fixation period (2125, 4125, or 7125 ms, 80/80/40 distribution across the 200 trials, pseudo-randomized), after which the next scene appeared. The study period was followed by a distractor task (i.e., solving simple mathematical problems, 1 min) and a rest period (3 min).

During the test period, participants viewed all scene images that were shown during the previous study period, intermixed with 100 novel scene images (i.e., a total of 300 scene images). Scenes were presented for 4 s each and were followed by a 6-point rating scale that required participants to indicate whether they recognized the scene as “old” or “new” (self-paced; the scale ranged from (1) “very sure old” to (6) “very sure new”), and a fixation period until the next trial started (2125, 4125, or 7125 ms, 80/80/40 distribution across the 300 trials, pseudo-randomized). The test period was divided into 2 blocks separated by a short break.

Study 2 (Vienna study):
The recognition memory task consisted of two study-test cycles (i.e., study1, test1, study2, test2). During each study period, participants were instructed to memorize 48 scene and 48 face images. An image was shown for 3 s, during which participants could freely view the image and was followed by a fixation cross (inter-trial-interval 2-7 s, mean 5 s). Across both study-test cycles, participants studied 192 images.

After each study period, participants completed a test period (i.e., the immediate test) where the 96 previously viewed (“old”) images were pseudo-randomly interleaved with 48 novel (“new”) images. As above, each image was presented for 3 s, followed by a 4-point rating scale that prompted participants to indicate whether they recognized the image as “old” or “new”, ranging from “very sure old” to “very sure new” (duration 2 s). The next trial started after an inter-trial-interval during which a fixation cross was presented on the computer screen (duration 2-7 s, mean 5 s).

The analysis pertained solely to the scene images to enable a more direct comparison between discovery and validation studies.

Behavioral performance measures

Recognition memory performance was quantified as d-prime, calculated as the difference between hit and false alarm rates [z(hits) – z(false alarms)].

Acquisition

Imaging type(s)

functional & structural

Field strength

3 Tesla

Sequence & imaging parameters

Study 1 (Donders study):
Imaging data were collected at the Donders Institute for Brain, Cognition and Behaviour (Nijmegen, The Netherlands), using a 3T Prisma Fit scanner (Siemens, Erlangen, Germany) equipped with a 32-channel head coil. We acquired on average 2456 (± 5.3) T2*-weighted blood oxygen level-dependent (BOLD) images during the study period of the recognition memory task, using the following echo-planar imaging (EPI) sequence: repetition time (TR) = 657 ms, echo time (TE) = 30.8 ms, multi-band acceleration factor = 8, 72 axial slices, interleaved acquisition, field of view (FoV) = 174 × 174 mm, 72 × 72 matrix, flip angle = 53°, slice thickness = 2.4 mm, no slice gap, voxel size = 2.4 mm isotropic. The structural image was acquired using a standard magnetization-prepared rapid gradient-echo (MPRAGE) sequence with the following parameters: TR = 2300 ms, TE = 3.03 ms, FoV = 256 × 256 mm, flip angle = 8°, voxel size = 1 mm isotropic.

Study 2 (Vienna study):
Imaging data were collected at the Neuroimaging Center of the University of Vienna, using a 3T Skyra MR- Scanner (Siemens, Erlangen, Germany) equipped with a 32-channel head coil. On average, we acquired 396.77 (± 7.24 SD) T2*-weighted blood oxygenation level-dependent (BOLD) images during each of the two study periods and 732.54 (± 9.10 SD) BOLD images during the two immediate test periods of the recognition memory task. We used the following partial-volume echo-planar imaging (EPI) sequence: TR = 2029 ms; TE = 30 ms; number of slices = 30 axial slices; slice order = interleaved acquisition; FoV = 216 mm; flip angle = 90°; slice thickness = 3 mm; in-plane resolution = 2 x 2 mm, using parallel imaging with GRAPPA acceleration factor of 2. Slice orientation was parallel to the line connecting the anterior and posterior commissure (AC-PC alignment), with a 10° rotational shift upwards. The T1-weighted structural image was acquired using a standard magnetization-prepared rapid gradient-echo (MPRAGE) sequence with the following parameters: TR = 2300 ms; TE = 2.43 ms; FoV = 240 mm; flip angle = 8°; voxel size = 0.8 mm isotropic. We additionally acquired a T2-weighted structural image used to delineate the entorhinal cortex. A turbo-spin-echo (TSE) Sampling Perfection with Application optimized Contrasts was applied using different flip angle Evolution (SPACE) sequence with the following parameters: TR = 3.2 s; TE = 564 ms; FoV = 256 mm, voxel size = 0.8 mm isotropic, slices were oriented perpendicular to the long axis of the hippocampus.

Due to the local proximity to air-filled cavities, entorhinal cortices are susceptible to image distortions. To ameliorate this effect, we collected 30 images with the same functional sequence but with a reversed phase-encoding direction (thus, stretching potential image distortions into the opposite direction). Additionally, we acquired 10 whole-brain EPI images to facilitate the co-registration of anatomical EC masks to the partial-volume EPI images with the following parameters: TR = 2.832 s, TE = 30 ms, number of slices = 42 axial slices, slice order = interleaved acquisition, FoV = 216 mm, flip angle = 90°, slice thickness = 3 mm, in-place resolution = 2 x 2 mm, using parallel imaging with a GRAPPA acceleration factor of 2. As above, slices were oriented parallel to the AC-PC line with a 10° rotational shift upwards.

Area of acquisition

Study 1 (Donders study):
whole-brain

Study 2 (Vienna study):
partial-volume and whole-brain, slices oriented parallel to line connecting anterior and posterior commissure (AC-PC alignment), 10° rotational shift upwards

Diffusion MRI ☐ Used ☒ Not used

Preprocessing

Preprocessing software

Study 1 (Donders study):
The fMRI data were processed with SPM8 in combination with MATLAB (The Mathworks, Natick, MA, USA). The first 12 volumes were excluded to allow for T1-equilibration. The remaining volumes (of both the study and test periods) were realigned to the mean image. The structural scan was co-registered to the mean functional image and was segmented into grey matter, white matter, and cerebrospinal fluid using the “New Segmentation” algorithm. All images (functional and structural) were then spatially normalized to the Montreal Neurological Institute (MNI) EPI template using Diffeomorphic Anatomical Registration Through Exponentiated Lie Algebra (DARTEL; Ashburner, 2007a), and functional images were further smoothed with a 3D Gaussian kernel (6 mm full-width at half-maximum, FWHM).

Study 2 (Vienna study):
The fMRI data were processed using SPM12 (https://www.fil.ion.ucl.ac.uk/spm/) in combination with MATLAB (The Mathworks, Natick, MA, USA, R2020b). Structural and functional scans were manually AC-PC corrected. The first six functional volumes were then excluded to allow for T1-equilibration. The remaining volumes were slice-time-corrected to the middle slice and spatially realigned to the mean functional image (across both study-test cycles). FSL’s “topup” command (FMRIB Software Library; https://fsl.fmrib.ox.ac.uk/fsl/fslwiki/topup; Jenkinson et al., 2012) was applied to correct potential image distortions. Specifically, the mean functional image was calculated based on the 30 functional volumes (with the reversed phase-encoding direction) and was used to estimate and correct susceptibility-induced distortions. To analyze grid-like codes in subject-native space, we refrained from normalizing the data. Functional images were smoothed with a 3D Gaussian kernel (5 mm FWHM).

Normalization

Functional and structural images were normalized to the MNI EPI (MNI-152) template using a non-linear approach: Diffeomorphic Anatomical Registration Through Exponentiated Lie Algebra (DARTEL; Ashburner, 2007).

Normalization template

MNI-152

Noise and artifact removal

Volumes were realigned to the mean functional image.

Study 2 (Donders study): Potential image distortions were corrected by applying FSL's "topup" command (s. above). The mean functional images was calculated based on 30 additional functional volumes (with the reversed phase-encoding direction), used to estimate and correct susceptibility-induced distortions.

Volume censoring

No volume censoring

Statistical modeling & inference

Model type and settings

Analysis of grid-like codes:

Grid-like codes were analyzed using the openly available Grid Code Analysis Toolbox (GridCAT, software version 1.0.4, https://www.nitrc.org/projects/gridcat; (Stangl et al., 2017) which is based on the procedures developed by Doeller et al. (2010).

Saccades during the study period of the recognition memory task were defined as events-of-interest. We then leveraged the General Linear Model (GLM) to model the BOLD response time-locked to saccade onsets. All saccades were estimated with stick functions (duration = 0 seconds) and were convolved with the SPM default canonical hemodynamic response function (HRF). To account for noise due to head movement, we included the six realignment parameters, their first derivatives, and the squared first derivatives into the design matrix. A high-pass filter with a cutoff at 128 s was applied.

Analysis of grid-like codes progressed in two steps pertaining to estimating and testing individual grid orientations. We partitioned the data into two equally-sized data halves (i.e., corresponding to two separate regressors that contained the saccades of the estimation or test data sets, respectively). During step 1 (GLM 1), saccade-related activity of the estimation data set (i.e., the first regressor) was modulated by the respective saccade direction. This was calculated as saccade angle (αt) relative to a predefined reference point and was modeled using two parametric modulators, $\sin(\alpha t*6)$ and $\cos(\alpha t*6)$, that converted directional information into 60° space, reflecting the hypothesized 6-fold rotational symmetry in the fMRI signal (presumably due to the firing patterns of underlying grid cells). The voxel-wise beta estimates, $\beta 1$ and $\beta 2$, of the two parametric modulators were then extracted, and the mean grid orientation within the respective ROI was calculated using $\arctan[\text{mean}(\beta 1)/\text{mean}(\beta 2)]/6$ (i.e., converting directional information back into 360° space). During step 2, the estimated grid orientations were then tested in a second GLM (GLM 2) that was virtually identical to the abovementioned model, but with the exception that the saccades within the first regressor (i.e., the estimation data set) were unmodulated, while the saccades in the second regressor (i.e., the test data set) were parametrically modulated by the difference between the respective saccade angle (αt) and the individual ROI-based grid orientation (ϕ) using $\cos[6*(\alpha t-\phi)]$. In other words, a smaller difference between αt and ϕ should result in increased grid-like codes since the saccade direction is aligned with the individual grid orientation. The beta values from the parametric modulator were then extracted for all voxels within the ROI and were averaged to produce the mean amount of grid-like coding.

Whole-brain analysis using a mass univariate approach (Donders study only):

We performed additional analyses to test whether the activity of entorhinal grid-like codes modulated voxel-wise changes in whole-brain activation. The amount of grid-like coding of each saccade was taken from the results of GLM 2 (this GLM had tested the previously estimated grid orientations in the second half of the data). To obtain grid-like codes for the first half of the data, we repeated the analysis but reversed the partitioning of the estimation/test data sets (i.e., we estimated grid orientations on the second data half and tested them on the first data half). Saccade-based grid-like codes were then extracted from the parametric modulation regressor (i.e., relying on the difference between each saccade's translational direction and the mean grid orientation of the participant, whereby a smaller difference should be associated with a stronger grid-like signal within the entorhinal cortex). We then averaged the amount of grid-like coding of all saccades within a trial, producing a trial-wise value for grid-like coding.

Next, to be able to perform a group-based analysis, we used the normalized, standard-space data and created a separate GLM (GLM 3). This model contained a single task regressor that captured all scene trials that were presented during the study period (modeled with a boxcar function, duration 4 s). This regressor was parametrically modulated with trial-wise grid-like coding. As above, GLM 3 included the six realignment parameters, their first derivatives, and the squared first derivatives into the design matrix. A high-pass filter with a cutoff at 128 s was applied.

Effect(s) tested

Analysis of grid-like codes:
ROI-based grid-like code data were analyzed using a set of Wilcoxon-tests in R (software version 4.3.0; https://www.r-project.org; R stats version 3.6.2). We hypothesized that significant grid-like coding in the entorhinal cortex should be associated with a 6-fold rotational symmetry of the fMRI signal in the entorhinal cortex. The choice of the statistical test thus reflected an a priori expectation, which is why we adopted an α-level of 0.05 (one-tailed). Effect sizes were calculated as Cohen's d.

Whole-brain analysis (Donders study only):
We contrasted the parametric modulation regressors that captured the trial-wise fluctuations in entorhinal grid-like coding against baseline (entorhinal grid-like coding during scene > implicit baseline) and tested for group effects by submitting the individual contrast images to a one-sample t-test.

Specify type of analysis: ☐ Whole brain ☐ ROI-based ☒ Both

Study 1 (Donders study): Entorhinal cortex

Anatomical location(s)

For the analysis of grid-like codes, left and right posterior medial entorhinal cortex masks were based on Maass et al., 2015. Masks were binarized and co-registered to the mean functional image of one participant (Maass, bilateral entorhinal cortex: 25 voxels, left entorhinal cortex: 14 voxels, right entorhinal cortex: 18 voxels). To validate the quality of the co-registration, the overlap between each mask and the corresponding (co-registered) structural and mean functional image was visually assessed for each participant.

Control Regions:
To test for potential grid-like codes in control regions, we defined additional ROIs that are known to be involved in memory, visuo-spatial processing, and oculomotor control, but for which no significant grid-like codes have been detected. This included the hippocampus, anterior thalamus, frontal eye fields, and visual cortex. The hippocampus and visual cortex were defined based on bilateral anatomical masks of the Automatic Anatomical Labeling (AAL) atlas (Tzourio-Mazoyer et al., 2002; hippocampus = 1148 voxels, visual cortex = 1860 voxels). To delineate the anterior thalamus, we used the stereotactic mean anatomical atlas provided by Krauth and colleagues (Krauth et al., 2010; © University of Zurich and ETH Zurich, Axel Krauth, Rémi Blanc, Alejandra Poveda, Daniel Jeanmonod, Anne Morel, Gábor Székely), which is based on histological, cytoarchitectural features defined ex vivo (Morel, 2007). We specified the anterior thalamus by combining the bilateral anterior dorsal, -medial, and -ventral nucleus masks (59 voxels). The frontal eye fields were defined by contrasting memory-related activity during scene encoding across all participants (later remembered > later forgotten). The resulting cluster peak coordinate (x = 43, y = 7, z = 29) was surrounded by a 10 mm sphere and was mirrored to create a bilateral ROI (320 voxels). See Supplementary information, Figure S2A).

Study 2 (Vienna study): Entorhinal cortex
Left and right entorhinal cortex masks were segmented using the Automatic Segmentation of Hippocampal Subfields algorithm (Yushkevich et al., 2015; ASHS, software version 1.0.0, https://sites.google.com/site/hipposubfields/) based on each participant's T1- and T2-weighted, high-resolution structural image. Masks were binarized and transformed into the subject-native space of the (partial-volume) functional images. To facilitate co-registration (which can be hampered by the partial-volume field-of-view), we progressed in several steps: First, participants' T2-weighted structural scan (along with the segmented left and right entorhinal cortex masks) was co-registered to align with the mean functional image (based on the 10 whole-brain functional images we acquired). Second, the mean (whole-brain) functional image (along with the co-registered T2 image and the entorhinal cortex masks) was co-registered to the mean (partial-volume) functional image. The overlap between each entorhinal cortex mask and the corresponding (co-registered) structural and functional data was visually inspected for each participant.

Due to its location close to the lateral ventricle, the entorhinal cortex can be associated with a lower signal-to-noise ratio. To bypass this issue, only voxels that exceeded a signal-to-noise threshold of 0.8 were examined, mainly leading to the exclusion of voxels along the anterior-medial entorhinal cortex border. Participants with less than 14 voxels in the (left or right) entorhinal cortex mask were excluded from the analyses. Consequently, in alignment with the ROI from the discovery study, analyses were focused on the posterior-medial entorhinal cortex and were based on a final sample of 20 participants (mean ± SEM; left entorhinal cortex, 19.85 ± 1.09 voxels, right entorhinal cortex, 20.35 ± 1.19 voxels).

Statistic type for inference

(See Eklund et al. 2016)

Significance for the whole-brain fMRI analysis was assessed using cluster-inference with a cluster-defining threshold of $p < 0.001$ and a cluster-probability of $p < 0.05$ family-wise error (FWE) corrected for multiple comparisons. The corrected cluster size (i.e., the spatial extent of a cluster that is required in order to be labeled as significant) was calculated using the SPM extension "CorrClusTh.m" and the Newton-Raphson search method (script provided by Thomas Nichols, University of Warwick, United Kingdom, and Marko Wilke, University of Tübingen, Germany; http://www2.warwick.ac.uk/fac/sci/statistics/staff/academic-research/nichols/scripts/spm/).

Correction

Family-wise error (FWE) correction for multiple comparisons.

Models & analysis

n/a	Involved in the study
☒	☐ Functional and/or effective connectivity
☒	☐ Graph analysis
☒	☐ Multivariate modeling or predictive analysis
